# Supplementary material for: Hot spot prediction in protein-protein interactions by an ensemble system
Source: BMC Syst Biol. 2018 Dec 31;12(Suppl 9):132. doi: 10.1186/s12918-018-0665-8 (PMC6311905; doi:10.1186/s12918-018-0665-8)
Supplement: Supplementary file 2 — All relevant MATLAB code. A simple Matlab implement of our predictor. (ZIP 19,742 kb) [file 12918_2018_665_MOESM2_ESM.zip › Additional file 2/Read ASA/ASA website/dbMPIKT/4X4ME.html]

 NetSurfP - Protein Surface Accessibility and Secondary Structure Predictions

  

|  |  |  |
| --- | --- | --- |
|  |  | NetSurfP - Protein Surface Accessibility and Secondary Structure Predictions  Technical University of Denmark |

  


---

```
# For publication of results, please cite:
# A generic method for assignment of reliability scores applied to solvent accessibility predictions.
# Bent Petersen, Thomas Nordahl Petersen, Pernille Andersen, Morten Nielsen and Claus Lundegaard
# BMC Structural Biology 2009, 9:51 doi:10.1186/1472-6807-9-51
#
# Column 1: Class assignment - B for buried or E for Exposed - Threshold: 25% exposure, but not based on RSA
# Column 2: Amino acid
# Column 3: Sequence name
# Column 4: Amino acid number
# Column 5: Relative Surface Accessibility - RSA
# Column 6: Absolute Surface Accessibility
# Column 7: Z-fit score for RSA prediction
# Column 8: Probability for Alpha-Helix
# Column 9: Probability for Beta-strand
# Column 10: Probability for Coil
E K  4X4ME                  1    0.677 139.279   1.588   0.003   0.003   0.994
B A  4X4ME                  2    0.120  13.235  -3.478   0.004   0.197   0.799
E V  4X4ME                  3    0.437  67.167   1.467   0.001   0.900   0.099
B I  4X4ME                  4    0.105  19.481  -0.246   0.001   0.959   0.040
E K  4X4ME                  5    0.375  77.117   0.992   0.001   0.959   0.040
B L  4X4ME                  6    0.215  39.440   0.211   0.001   0.900   0.099
E Q  4X4ME                  7    0.447  79.852   0.692   0.004   0.514   0.481
E P  4X4ME                  8    0.252  35.702  -1.024   0.005   0.045   0.951
E P  4X4ME                  9    0.706 100.125   0.109   0.005   0.015   0.979
E W  4X4ME                 10    0.662 159.139  -0.615   0.004   0.138   0.858
B V  4X4ME                 11    0.219  33.691   0.585   0.004   0.514   0.481
E S  4X4ME                 12    0.531  62.280   1.023   0.018   0.846   0.136
B V  4X4ME                 13    0.088  13.495   0.500   0.011   0.918   0.071
E F  4X4ME                 14    0.398  79.778   0.988   0.003   0.718   0.279
E Q  4X4ME                 15    0.549  98.087   0.885   0.004   0.197   0.799
E E  4X4ME                 16    0.704 123.024  -0.422   0.005   0.015   0.979
E E  4X4ME                 17    0.447  78.178   1.105   0.004   0.085   0.910
E S  4X4ME                 18    0.543  63.651   1.570   0.004   0.616   0.381
B V  4X4ME                 19    0.052   8.023   0.486   0.001   0.959   0.040
E T  4X4ME                 20    0.337  46.770   2.002   0.001   0.959   0.040
B L  4X4ME                 21    0.038   7.013   0.394   0.001   0.959   0.040
E H  4X4ME                 22    0.343  62.410   1.885   0.001   0.959   0.040
B C  4X4ME                 23    0.016   2.204   1.103   0.001   0.900   0.099
E E  4X4ME                 24    0.400  69.845   1.195   0.001   0.959   0.040
B V  4X4ME                 25    0.069  10.682  -1.084   0.001   0.900   0.099
E P  4X4ME                 26    0.554  78.627   0.186   0.004   0.514   0.481
E H  4X4ME                 27    0.569 103.483  -2.058   0.004   0.138   0.858
E L  4X4ME                 28    0.569 104.221  -0.854   0.005   0.045   0.951
E P  4X4ME                 29    0.360  51.098  -0.750   0.005   0.015   0.979
E G  4X4ME                 30    0.588  46.307  -0.257   0.004   0.085   0.910
E S  4X4ME                 31    0.439  51.451  -0.806   0.005   0.262   0.733
B S  4X4ME                 32    0.267  31.316   0.269   0.004   0.616   0.381
E S  4X4ME                 33    0.362  42.380   1.338   0.001   0.900   0.099
B T  4X4ME                 34    0.038   5.257   0.711   0.001   0.959   0.040
B Q  4X4ME                 35    0.242  43.203   1.839   0.001   0.959   0.040
B W  4X4ME                 36    0.037   8.778   0.123   0.001   0.959   0.040
B F  4X4ME                 37    0.201  40.401   1.346   0.001   0.959   0.040
B L  4X4ME                 38    0.191  34.954   0.834   0.002   0.816   0.182
E N  4X4ME                 39    0.664  97.239   0.169   0.005   0.015   0.979
E G  4X4ME                 40    0.741  58.340  -0.668   0.005   0.045   0.951
E T  4X4ME                 41    0.634  87.922   0.566   0.002   0.816   0.182
E A  4X4ME                 42    0.513  56.577   0.696   0.001   0.900   0.099
B I  4X4ME                 43    0.149  27.620   0.538   0.021   0.756   0.223
E Q  4X4ME                 44    0.560  99.980   0.698   0.004   0.514   0.481
E T  4X4ME                 45    0.464  64.315  -0.556   0.005   0.336   0.660
E S  4X4ME                 46    0.646  75.676  -1.160   0.004   0.138   0.858
E T  4X4ME                 47    0.650  90.169  -0.885   0.004   0.085   0.910
E P  4X4ME                 48    0.411  58.264   0.021   0.005   0.336   0.660
E T  4X4ME                 49    0.372  51.596   0.784   0.002   0.816   0.182
B Y  4X4ME                 50    0.048  10.322   0.626   0.001   0.959   0.040
E H  4X4ME                 51    0.376  68.303   1.365   0.001   0.959   0.040
B I  4X4ME                 52    0.040   7.455  -0.101   0.001   0.900   0.099
E T  4X4ME                 53    0.470  65.244   1.158   0.003   0.718   0.279
E S  4X4ME                 54    0.576  67.542   0.613   0.004   0.514   0.481
B A  4X4ME                 55    0.139  15.274   0.511   0.005   0.336   0.660
E S  4X4ME                 56    0.488  57.205   0.888   0.004   0.420   0.576
E E  4X4ME                 57    0.574 100.225   0.324   0.004   0.514   0.481
E D  4X4ME                 58    0.627  90.351  -0.308   0.004   0.197   0.799
E D  4X4ME                 59    0.285  41.054  -0.836   0.004   0.085   0.910
E S  4X4ME                 60    0.540  63.276  -0.339   0.004   0.138   0.858
B G  4X4ME                 61    0.177  13.961  -1.793   0.003   0.718   0.279
E E  4X4ME                 62    0.403  70.474   1.883   0.001   0.900   0.099
B Y  4X4ME                 63    0.043   9.168   0.596   0.001   0.959   0.040
E R  4X4ME                 64    0.277  63.341   2.016   0.001   0.959   0.040
B C  4X4ME                 65    0.016   2.232   1.162   0.001   0.959   0.040
E Q  4X4ME                 66    0.294  52.473   1.609   0.001   0.959   0.040
B R  4X4ME                 67    0.055  12.572  -0.230   0.001   0.959   0.040
E G  4X4ME                 68    0.473  37.209   0.204   0.002   0.816   0.182
E L  4X4ME                 69    0.334  61.229  -0.704   0.004   0.420   0.576
E S  4X4ME                 70    0.598  70.027  -0.917   0.004   0.138   0.858
E G  4X4ME                 71    0.609  47.913  -0.689   0.005   0.262   0.733
E R  4X4ME                 72    0.470 107.653  -0.580   0.004   0.420   0.576
E S  4X4ME                 73    0.395  46.259  -0.728   0.004   0.420   0.576
E D  4X4ME                 74    0.481  69.240   0.261   0.003   0.718   0.279
E P  4X4ME                 75    0.447  63.387   1.195   0.001   0.900   0.099
B I  4X4ME                 76    0.082  15.170   0.285   0.001   0.959   0.040
E Q  4X4ME                 77    0.361  64.421   1.480   0.001   0.959   0.040
B L  4X4ME                 78    0.051   9.356   0.726   0.001   0.959   0.040
E E  4X4ME                 79    0.304  53.021   0.963   0.001   0.900   0.099
B V  4X4ME                 80    0.044   6.794   0.179   0.001   0.900   0.099
E H  4X4ME                 81    0.345  62.828   0.376   0.003   0.718   0.279
E R  4X4ME                 82    0.359  82.303   0.239   0.005   0.336   0.660
E G  4X4ME                 83    0.436  34.313   0.153   0.018   0.088   0.893
B W  4X4ME                 84    0.261  62.867  -0.168   0.019   0.141   0.840
E L  4X4ME                 85    0.453  83.018  -0.244   0.023   0.655   0.322
E L  4X4ME                 86    0.309  56.578  -0.176   0.021   0.756   0.223
E L  4X4ME                 87    0.273  49.895   0.010   0.022   0.552   0.426
E Q  4X4ME                 88    0.552  98.641   0.014   0.005   0.336   0.660
E V  4X4ME                 89    0.262  40.331  -0.343   0.004   0.085   0.910
E S  4X4ME                 90    0.593  69.523  -0.512   0.018   0.047   0.935
E S  4X4ME                 91    0.655  76.731  -0.419   0.018   0.047   0.935
E R  4X4ME                 92    0.375  85.875   0.175   0.021   0.279   0.699
E V  4X4ME                 93    0.503  77.342   0.642   0.003   0.718   0.279
B L  4X4ME                 94    0.106  19.335   0.038   0.018   0.846   0.136
E T  4X4ME                 95    0.452  62.706   0.872   0.003   0.718   0.279
E E  4X4ME                 96    0.491  85.778   0.327   0.005   0.336   0.660
E G  4X4ME                 97    0.576  45.315  -1.241   0.005   0.045   0.951
E E  4X4ME                 98    0.505  88.223   0.753   0.004   0.085   0.910
E P  4X4ME                 99    0.504  71.489   1.385   0.004   0.616   0.381
B L  4X4ME                100    0.037   6.830   0.137   0.001   0.900   0.099
E A  4X4ME                101    0.339  37.380   1.953   0.001   0.959   0.040
B L  4X4ME                102    0.039   7.086   0.345   0.001   0.959   0.040
E R  4X4ME                103    0.296  67.715   1.636   0.001   0.959   0.040
B C  4X4ME                104    0.015   2.134   1.046   0.001   0.900   0.099
E H  4X4ME                105    0.407  73.997   1.493   0.001   0.959   0.040
B A  4X4ME                106    0.053   5.841  -1.212   0.001   0.900   0.099
E W  4X4ME                107    0.556 133.766   0.312   0.004   0.420   0.576
E K  4X4ME                108    0.689 141.768  -1.735   0.004   0.085   0.910
E D  4X4ME                109    0.630  90.783  -1.607   0.005   0.015   0.979
E K  4X4ME                110    0.468  96.247  -0.645   0.005   0.015   0.979
E L  4X4ME                111    0.531  97.244  -0.261   0.004   0.085   0.910
E V  4X4ME                112    0.361  55.547  -0.101   0.005   0.262   0.733
B Y  4X4ME                113    0.178  38.017  -0.314   0.004   0.514   0.481
E N  4X4ME                114    0.344  50.391   0.882   0.002   0.816   0.182
B V  4X4ME                115    0.039   5.979   0.468   0.001   0.959   0.040
B L  4X4ME                116    0.209  38.250   1.500   0.001   0.959   0.040
B Y  4X4ME                117    0.050  10.664   0.659   0.001   0.959   0.040
B Y  4X4ME                118    0.214  45.668   1.138   0.001   0.959   0.040
B R  4X4ME                119    0.235  53.815   0.863   0.002   0.816   0.182
E N  4X4ME                120    0.613  89.772  -0.547   0.005   0.015   0.979
E G  4X4ME                121    0.702  55.216  -1.714   0.018   0.019   0.964
E K  4X4ME                122    0.573 117.928   0.023   0.002   0.816   0.182
E A  4X4ME                123    0.522  57.469   0.117   0.002   0.816   0.182
B F  4X4ME                124    0.146  29.302   0.472   0.023   0.655   0.322
E K  4X4ME                125    0.547 112.559   0.370   0.021   0.451   0.528
E F  4X4ME                126    0.425  85.318  -1.685   0.004   0.420   0.576
E F  4X4ME                127    0.466  93.506  -0.853   0.005   0.262   0.733
E H  4X4ME                128    0.509  92.660  -1.265   0.004   0.138   0.858
E W  4X4ME                129    0.745 179.197  -0.523   0.004   0.085   0.910
E N  4X4ME                130    0.598  87.518  -1.090   0.004   0.085   0.910
B S  4X4ME                131    0.156  18.260  -0.877   0.005   0.262   0.733
E N  4X4ME                132    0.357  52.236   0.904   0.002   0.816   0.182
B L  4X4ME                133    0.032   5.914   0.162   0.001   0.959   0.040
E T  4X4ME                134    0.297  41.249   1.564   0.001   0.959   0.040
B I  4X4ME                135    0.037   6.789   0.151   0.001   0.900   0.099
E L  4X4ME                136    0.449  82.139   1.306   0.003   0.718   0.279
E K  4X4ME                137    0.575 118.319   0.893   0.004   0.514   0.481
B T  4X4ME                138    0.077  10.638   0.044   0.005   0.336   0.660
E N  4X4ME                139    0.440  64.387   1.053   0.005   0.336   0.660
E M  4X4ME                140    0.540 107.994   0.325   0.004   0.197   0.799
E S  4X4ME                141    0.652  76.426  -0.464   0.004   0.085   0.910
B H  4X4ME                142    0.209  37.963  -0.854   0.005   0.045   0.951
E S  4X4ME                143    0.483  56.561  -0.547   0.004   0.138   0.858
B G  4X4ME                144    0.187  14.678  -1.714   0.003   0.718   0.279
E T  4X4ME                145    0.424  58.809   1.812   0.001   0.900   0.099
B Y  4X4ME                146    0.046   9.809   0.647   0.001   0.959   0.040
E H  4X4ME                147    0.276  50.277   1.909   0.001   0.959   0.040
B C  4X4ME                148    0.015   2.078   1.387   0.001   0.959   0.040
E S  4X4ME                149    0.250  29.277   2.037   0.001   0.959   0.040
B G  4X4ME                150    0.039   3.061  -0.759   0.001   0.959   0.040
E M  4X4ME                151    0.401  80.240   0.555   0.001   0.959   0.040
B G  4X4ME                152    0.168  13.214  -0.928   0.004   0.616   0.381
E K  4X4ME                153    0.637 130.928  -0.428   0.004   0.085   0.910
E H  4X4ME                154    0.558 101.537  -1.315   0.004   0.085   0.910
E R  4X4ME                155    0.517 118.370  -0.904   0.005   0.262   0.733
E Y  4X4ME                156    0.556 118.710  -0.368   0.005   0.336   0.660
E T  4X4ME                157    0.398  55.203  -0.532   0.004   0.420   0.576
E S  4X4ME                158    0.453  53.127  -0.709   0.004   0.616   0.381
E A  4X4ME                159    0.417  45.953   0.226   0.004   0.616   0.381
E G  4X4ME                160    0.478  37.619   0.890   0.002   0.816   0.182
B I  4X4ME                161    0.068  12.561   0.099   0.001   0.959   0.040
E S  4X4ME                162    0.381  44.595   1.557   0.001   0.959   0.040
B V  4X4ME                163    0.030   4.550   0.308   0.001   0.959   0.040
E T  4X4ME                164    0.271  37.588   1.891   0.001   0.959   0.040
B V  4X4ME                165    0.024   3.658   0.879   0.001   0.959   0.040
E K  4X4ME                166    0.383  78.742   1.341   0.001   0.900   0.099
B E  4X4ME                167    0.199  34.713   0.263   0.002   0.816   0.182
E L  4X4ME                168    0.500  91.550   0.106   0.005   0.262   0.733
E F  4X4ME                169    0.318  63.762  -0.239   0.004   0.085   0.910
E P  4X4ME                170    0.582  82.628  -0.328   0.004   0.085   0.910
E A  4X4ME                171    0.413  45.502  -0.378   0.004   0.197   0.799
E P  4X4ME                172    0.306  43.421  -0.545   0.004   0.514   0.481
E V  4X4ME                173    0.345  53.026  -0.869   0.002   0.816   0.182
B L  4X4ME                174    0.271  49.675  -0.626   0.002   0.816   0.182
E T  4X4ME                175    0.463  64.163   0.034   0.021   0.756   0.223
E A  4X4ME                176    0.337  37.115  -0.365   0.005   0.336   0.660
E S  4X4ME                177    0.429  50.255  -0.614   0.004   0.085   0.910
E V  4X4ME                178    0.641  98.476  -0.844   0.018   0.047   0.935
E T  4X4ME                179    0.647  89.683  -1.132   0.018   0.088   0.893
E S  4X4ME                180    0.338  39.567   0.084   0.022   0.359   0.619
E P  4X4ME                181    0.528  74.895   0.492   0.003   0.718   0.279
B L  4X4ME                182    0.089  16.296   0.127   0.003   0.718   0.279
E L  4X4ME                183    0.438  80.289   0.817   0.003   0.718   0.279
E E  4X4ME                184    0.496  86.669   0.471   0.004   0.420   0.576
E G  4X4ME                185    0.638  50.234  -1.224   0.004   0.085   0.910
E T  4X4ME                186    0.460  63.857   0.598   0.004   0.085   0.910
E P  4X4ME                187    0.542  76.910   1.333   0.004   0.616   0.381
B V  4X4ME                188    0.037   5.672  -0.159   0.001   0.900   0.099
E T  4X4ME                189    0.365  50.653   1.934   0.001   0.959   0.040
B L  4X4ME                190    0.035   6.427   0.528   0.006   0.962   0.032
E S  4X4ME                191    0.334  39.157   1.739   0.011   0.918   0.071
B C  4X4ME                192    0.019   2.668   1.133   0.001   0.900   0.099
E E  4X4ME                193    0.385  67.242   1.321   0.001   0.900   0.099
B T  4X4ME                194    0.059   8.128  -0.792   0.001   0.900   0.099
E K  4X4ME                195    0.502 103.282   0.061   0.003   0.718   0.279
B L  4X4ME                196    0.195  35.705  -1.804   0.004   0.420   0.576
E L  4X4ME                197    0.471  86.185  -0.518   0.005   0.336   0.660
B L  4X4ME                198    0.174  31.804  -1.279   0.004   0.085   0.910
E Q  4X4ME                199    0.500  89.282  -0.207   0.005   0.045   0.951
E R  4X4ME                200    0.424  97.027  -1.119   0.018   0.047   0.935
E P  4X4ME                201    0.588  83.466  -2.165   0.018   0.047   0.935
E G  4X4ME                202    0.468  36.816  -1.162   0.004   0.138   0.858
B L  4X4ME                203    0.270  49.364  -0.446   0.004   0.420   0.576
E Q  4X4ME                204    0.404  72.172  -0.226   0.003   0.718   0.279
B L  4X4ME                205    0.120  22.027   0.035   0.002   0.816   0.182
B Y  4X4ME                206    0.237  50.690   0.660   0.001   0.900   0.099
B F  4X4ME                207    0.072  14.551   0.281   0.001   0.959   0.040
B S  4X4ME                208    0.232  27.226   0.843   0.001   0.959   0.040
B F  4X4ME                209    0.078  15.554  -0.205   0.001   0.959   0.040
E Y  4X4ME                210    0.249  53.147   0.394   0.002   0.816   0.182
E M  4X4ME                211    0.370  74.037   0.531   0.004   0.514   0.481
E G  4X4ME                212    0.677  53.248  -1.061   0.005   0.015   0.979
E S  4X4ME                213    0.756  88.556  -2.339   0.005   0.015   0.979
E K  4X4ME                214    0.497 102.253  -0.088   0.004   0.616   0.381
E T  4X4ME                215    0.541  75.023   0.469   0.003   0.718   0.279
B L  4X4ME                216    0.136  24.847   0.205   0.023   0.655   0.322
E R  4X4ME                217    0.511 117.065  -0.185   0.022   0.552   0.426
E G  4X4ME                218    0.458  36.029  -1.897   0.004   0.420   0.576
E R  4X4ME                219    0.504 115.347  -1.656   0.004   0.197   0.799
E D  4X4ME                220    0.533  76.863  -2.328   0.004   0.138   0.858
E T  4X4ME                221    0.602  83.539  -1.726   0.004   0.138   0.858
E S  4X4ME                222    0.504  59.116  -1.219   0.004   0.197   0.799
B S  4X4ME                223    0.164  19.221  -0.901   0.004   0.420   0.576
E E  4X4ME                224    0.340  59.485   0.676   0.002   0.816   0.182
B Y  4X4ME                225    0.038   8.078   0.137   0.001   0.959   0.040
E Q  4X4ME                226    0.296  52.955   1.355   0.001   0.959   0.040
B I  4X4ME                227    0.038   7.104   0.365   0.001   0.900   0.099
E L  4X4ME                228    0.461  84.336   1.349   0.003   0.718   0.279
E T  4X4ME                229    0.554  76.784   0.564   0.004   0.616   0.381
B A  4X4ME                230    0.125  13.731   0.146   0.005   0.336   0.660
E R  4X4ME                231    0.458 104.928   0.690   0.004   0.420   0.576
E R  4X4ME                232    0.571 130.782   0.147   0.005   0.336   0.660
E E  4X4ME                233    0.662 115.634  -0.123   0.004   0.138   0.858
B D  4X4ME                234    0.201  29.007  -1.120   0.004   0.085   0.910
E S  4X4ME                235    0.443  51.931  -0.659   0.004   0.197   0.799
B G  4X4ME                236    0.141  11.105  -1.577   0.002   0.816   0.182
E L  4X4ME                237    0.406  74.394   1.753   0.001   0.900   0.099
B Y  4X4ME                238    0.039   8.334   0.299   0.001   0.959   0.040
B W  4X4ME                239    0.258  61.977   1.968   0.001   0.959   0.040
B C  4X4ME                240    0.012   1.685   1.563   0.001   0.959   0.040
B E  4X4ME                241    0.226  39.482   2.003   0.001   0.959   0.040
B A  4X4ME                242    0.027   2.953  -0.213   0.001   0.959   0.040
E A  4X4ME                243    0.359  39.529   0.509   0.001   0.959   0.040
B T  4X4ME                244    0.092  12.733  -0.363   0.003   0.718   0.279
E E  4X4ME                245    0.576 100.610  -0.193   0.004   0.197   0.799
E D  4X4ME                246    0.418  60.234  -0.703   0.004   0.197   0.799
E G  4X4ME                247    0.432  33.959  -1.573   0.005   0.262   0.733
E N  4X4ME                248    0.560  81.999  -0.528   0.005   0.336   0.660
E V  4X4ME                249    0.374  57.530  -0.869   0.004   0.514   0.481
E L  4X4ME                250    0.509  93.253  -0.286   0.004   0.616   0.381
E K  4X4ME                251    0.494 101.616  -0.222   0.004   0.616   0.381
E R  4X4ME                252    0.405  92.676  -0.234   0.004   0.616   0.381
B S  4X4ME                253    0.302  35.371  -1.246   0.003   0.718   0.279
E P  4X4ME                254    0.333  47.224  -0.027   0.002   0.816   0.182
E E  4X4ME                255    0.417  72.780   0.902   0.001   0.900   0.099
B L  4X4ME                256    0.046   8.478   0.183   0.001   0.959   0.040
E E  4X4ME                257    0.389  67.871   1.498   0.001   0.959   0.040
B L  4X4ME                258    0.030   5.401   0.367   0.001   0.959   0.040
E Q  4X4ME                259    0.342  61.153   1.738   0.001   0.959   0.040
B V  4X4ME                260    0.046   7.086  -0.060   0.001   0.900   0.099
E L  4X4ME                261    0.513  93.857   0.722   0.004   0.616   0.381
E G  4X4ME                262    0.348  27.427  -0.573   0.021   0.279   0.699
E H  4X4ME                263    0.586 106.612  -0.603   0.019   0.141   0.840
E Q  4X4ME                264    0.589 105.267  -0.945   0.018   0.088   0.893
E Q  4X4ME                265    0.484  86.478  -0.747   0.018   0.047   0.935
E P  4X4ME                266    0.404  57.356  -1.079   0.018   0.088   0.893
E T  4X4ME                267    0.397  55.050  -0.883   0.019   0.141   0.840
E P  4X4ME                268    0.306  43.450  -0.901   0.019   0.141   0.840
B V  4X4ME                269    0.252  38.702  -1.555   0.004   0.197   0.799
E H  4X4ME                270    0.368  66.903  -1.394   0.005   0.262   0.733
E H  4X4ME                271    0.537  97.698  -1.115   0.004   0.197   0.799
E H  4X4ME                272    0.383  69.740  -0.555   0.004   0.197   0.799
E H  4X4ME                273    0.524  95.243  -0.951   0.004   0.138   0.858
E H  4X4ME                274    0.494  89.822  -1.055   0.018   0.088   0.893
E H  4X4ME                275    0.582 105.775  -2.067   0.003   0.003   0.994

Explain the output.  Go back.
```

---
